# Cdc13 at a crossroads of telomerase action

**DOI:** 10.3389/fonc.2013.00039

**Published:** 2013-02-28

**Authors:** Dmitri Churikov, Yves Corda, Pierre Luciano, Vincent Géli

**Affiliations:** Marseille Cancer Research Center, U1068 INSERM, UMR7258 CNRS, Aix-Marseille UniversityInstitut Paoli-Calmettes, Marseille, France

**Keywords:** telomeres, telomerase, replication, Cdc13, yKU70/80, replication protein A

## Abstract

Telomere elongation by telomerase involves sequential steps that must be highly coordinated to ensure the maintenance of telomeres at a proper length. Telomerase is delivered to telomere ends, where it engages single-strand DNA end as a primer, elongates it, and dissociates from the telomeres via mechanism that is likely coupled to the synthesis of the complementary C-strand. In *Saccharomyces cerevisiae*, the telomeric G-overhang bound Cdc13 acts as a platform for the recruitment of several factors that orchestrate timely transitions between these steps. In this review, we focus on some unresolved aspects of telomerase recruitment and on the mechanisms that regulate telomere elongation by telomerase after its recruitment to chromosome ends. We also highlight the key regulatory modifications of Cdc13 that promote transitions between the steps of telomere elongation.

## INTRODUCTION

Telomeres are nucleoprotein structures present at the ends of linear eukaryotic chromosomes with two essential functions. First, telomeres prevent recognition of the natural chromosomes ends as double-strand DNA breaks, which would lead to undesirable repair reactions with catastrophic consequences for the cell. Second, they facilitate and ensure complete replication of the chromosome ends that cannot be accomplished by semi-conservative DNA replication alone. The maintenance of repetitive telomeric DNA depends on telomerase, a ribonucleoprotein complex that uses its RNA template to elongate the telomere by addition of G-rich telomeric repeats to the terminal 3′ overhang (Gilson and Géli, 2007). To elongate telomeres, telomerase needs to be recruited to chromosome ends via a set of interactions with telomere-binding proteins. Then, the telomerase-extended single-strand DNA must be copied by DNA polymerase alpha to reconstitute the double-stranded telomere (Stewart et al., 2012). In the budding yeast *Saccharomyces cerevisiae*, the ends of chromosomes contain a 250–300 bp array of irregular TG_1__-__3_ repeats that arise due to incomplete reverse transcription of the telomerase RNA (TLC1) template and multiple registers of alignment of the chromosome terminus (which serves as a DNA primer) within the template region (Bairley et al., 2011). The 3′ single-stranded overhang is 12–14 nucleotides-long throughout most of the cell cycle except in late S-phase when longer overhangs are detected (Wellinger and Zakian, 2012). A single protein, Cdc13, binds telomeric single-stranded DNA with high affinity and sequence specificity and acts as a platform for the recruitment of different factors that in turn regulate protection of telomeric C-strand, recruitment of telomerase, and C_1__-__3_A strand re-synthesis by DNA polymerase α (Giraud-Panis et al., 2010).

In human cells, the function of Cdc13 is carried out by two different complexes, the POT1–TPP1 and the CTC1–STN1–TEN1 (CST; Price et al., 2010). The telomere-end binding complex POT1–TPP1 functions in both telomere protection and telomerase recruitment (Xin et al., 2007; Abreu et al., 2010; Tejera et al., 2010). The recruitment of telomerase at chromosome ends is mediated by an interaction between a surface-exposed loop within the oligosaccharide binding (OB)-fold of TPP1 and the telomerase catalytic subunit TERT (Zaug et al., 2010; Nandakumar et al., 2012; Sexton et al., 2012; Zhong et al., 2012). In addition, the POT1–TPP1 complex increases telomerase processivity during telomere extension (Wang et al., 2007; Latrick and Cech, 2010). Mammalian CST also binds telomeric single-stranded DNA and facilitates DNA replication at telomeres (Miyake et al., 2009; Surovtseva et al., 2009; Gu et al., 2012; Huang et al., 2012; Stewart et al., 2012), but it does not have a bona fide capping function as it does in budding yeast. On one end, the human CST negatively controls telomerase elongation by binding to telomerase-extended telomeres thereby limiting telomerase action (Chen et al., 2012). On the other end, the CST keeps telomere in check by limiting nuclease-mediated 3′ overhangs through stimulation of the “fill in” synthesis (Chai et al., 2006; Giraud-Panis et al., 2010; Wu et al., 2012). Therefore, in both organisms, yeast and humans, the CST complex limits 3′ overhangs and inhibits telomere elongation by telomerase while Cdc13–Est1 and the POT–TPP1 both play a critical role in recruiting telomerase to chromosome ends (Evans and Lundblad, 2000; Zhong et al., 2012).

In this review, we will revisit the model of telomerase recruitment and elongation in *S. cerevisiae* in the light of the most recent findings and discuss the multiple roles of Cdc13.

## CDC13 AT A CROSSROADS

The Cdc13 associates with Ten1 and Stn1 to form the CST complex with essential capping function. Ten1 plays a critical role in capping telomeres since it prevents the accumulation of aberrant single-stranded telomere DNA by limiting the Cdk1/Exo1-dependent degradation of the telomeric 5′ end (Grandin et al., 1997; Xu et al., 2009). Moreover, Ten1 increases the telomeric DNA-binding activity of Cdc13p to negatively regulate telomere length (Qian et al., 2009). This inhibition is consistent with the ability of Cdc13 to exert an inhibitory effect on primer extension by telomerase *in vitro* (Zappulla et al., 2009). Stn1 participates in the capping function of the CST by interacting via its N-terminus with Ten1 (Puglisi et al., 2008) and by stimulating the action of the DNA polymerase alpha-primase complex (Qi and Zakian, 2000; Grossi et al., 2004). In addition, Stn1 negatively regulates telomerase action by competing with Est1 for binding to Cdc13 (Grandin et al., 2000; Chandra et al., 2001). This notion is consistent with the discovery of *cdc13* alleles that result in telomerase-dependent extensive elongation of the telomeric G-strand that can be suppressed by overexpressing Stn1 (Chandra et al., 2001). In agreement with these results, cells lacking the Stn1 C-terminus display a strong telomere elongation phenotype even in the absence of Tel1 function (Puglisi et al., 2008). These results were further strengthened by the finding that Cdc13 post-translational modifications regulate its interaction with Est1 and Stn1–Ten1 (see further).

## TELOMERASE RECRUITMENT

Recruitment of telomerase to telomeres in budding yeast has been a subject of many excellent reviews (Hug and Lingner, 2006; Bianchi and Shore, 2008; Grandin and Charbonneau, 2008; DeZwaan and Freeman, 2010; Li, 2011; Dewar and Lydall, 2012; Wellinger and Zakian, 2012). Currently, telomerase recruitment can be best described as a cell cycle regulated process that begins with the nuclear import of the Yku-bound TLC1 (Gallardo et al., 2008). Yku80 subunit of the Yku ring binds a specific stem-loop region of TLC1 RNA (Peterson et al., 2001). Once delivered into nucleoplasm, TLC1 associates with Est2 at some point to form the core telomerase while retaining the interaction with Yku which is one of the most abundant nuclear proteins with high affinity for DNA ends. Occasionally, Yku-bound telomerase can encounter telomere ends. Such encounters were proposed to result in Yku engaging in DNA binding and thus releasing TLC1 RNA, since Yku has higher affinity for the former (Gallardo et al., 2011; Lopez et al., 2011; Pfingsten et al., 2012). Following this view, Yku could target telomerase to telomere terminus, but it cannot retain association with it at the site. For this reason, additional factors are required *in vivo* to anchor telomerase at the terminus. One important factor that serves this purpose is Est1 which abundance peaks in late S/G2-phase (Taggart et al., 2002; Osterhage et al., 2006). Est1 interacts with both a stem-bulge region in TLC1 and G-overhang bound Cdc13 which makes it an ideal bridging molecule (Evans and Lundblad, 1999; Pennock et al., 2001; Seto et al., 2002; Bianchi et al., 2004; Zappulla and Cech, 2004; Chan et al., 2008; Wu and Zakian, 2011). Est1 also interacts directly with Est3, another essential telomerase-associated protein (Hughes et al., 2000; Tuzon et al., 2011). The association of Est1 with Cdc13 at the expense of the Cdc13–Stn1 is partially controlled by cell cycle regulated phosphorylation of Cdc13 on T308 by Cdk1 (Li et al., 2009), but it is likely that multiple phosphorylation events act redundantly to control the protein interactions mediated by Cdc13 (Wu et al., 2012). Another modification, Cdc13 SUMOylation on Lys909 located in the Stn1-binding domain, favors the interaction between Cdc13 and Stn1. Consistently, the *K909R* mutation that abolishes Cdc13 SUMOylation leads to telomere lengthening (Hang et al., 2011). Because Cdc13 SUMOylation peaks in early to mid S-phase, it is likely that this modification antagonizes CDK1-dependant phosphorylation of T308 and restrains telomerase action before DNA replication is completed.

Binding of the telomerase complex is promoted at Rif2-depleted short telomeres by the MRX complex (Mre11, Rad50, and Xrs2) that recruits Tel1 to short telomeres via specific interaction with a conserved motif in the Xrs2 C-terminus (Nakada et al., 2003; McGee et al., 2010). At the same time, MRX complex (along with Sae2) participates in the resection of telomeric C-strand (Larrivée et al., 2004; Bonetti et al., 2009). The requirement for Tel1 in telomerase recruitment specifically to short telomeres has been well documented (Teixeira et al., 2004; Goudsouzian et al., 2006; Arneric and Lingner, 2007; Bianchi and Shore, 2007; Chang et al., 2007; Hector et al., 2007; Sabourin et al., 2007). However, it remains unclear whether Tel1 functions directly by phosphorylating specific targets at telomeres which promote telomerase recruitment, and/or indirectly by stimulating resection of the C-strand (by MRX–Sae2) and thus generating a longer substrate for telomerase recruitment (discussed in Gao et al., 2010).

## STABILIZATION OF TELOMERASE AT TELOMERES

**FIGURE 1:**
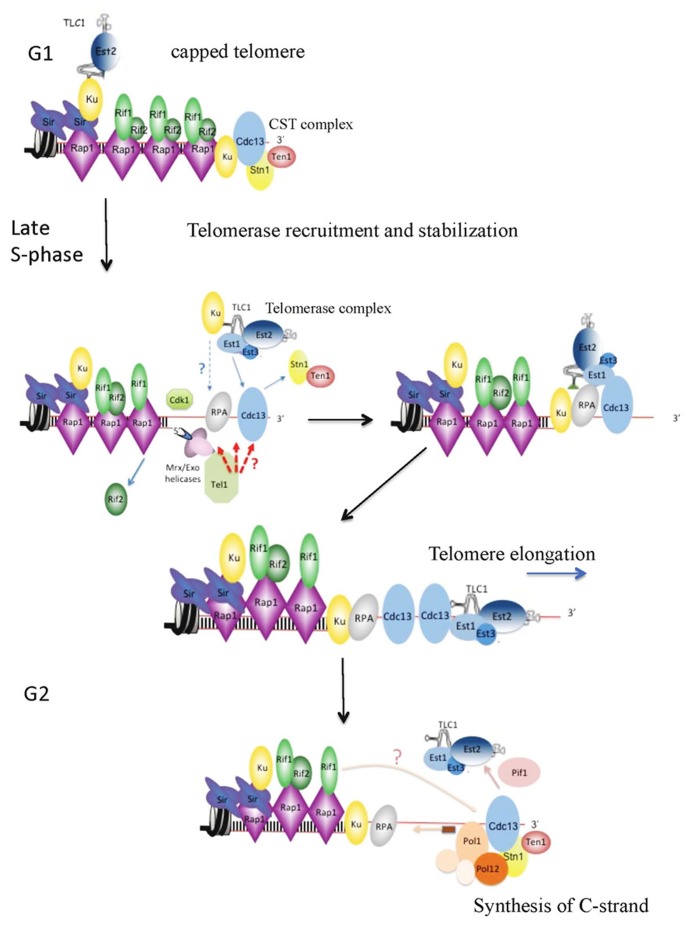
**Telomere elongation by telomerase in *Saccharomyces cerevisiae.*** G1-phase: telomeres are capped by the CST complex and by the yKu70/80 heterodimer. Telomerase recruitment is likely to begin with the nuclear import of the Yku-bound TLC1 and the subsequent association of Est2 and of its subunits to the TLC1 scaffold. Late S-phase: short telomeres are preferentially bound by the MRX complex and resected by two distinct but partially complementary pathways of nucleolytic resection. At this stage yKu may help to tether the telomerase holoenzyme to telomere ends. Productive recruitment of the telomerase holoenzyme will be ensured by the interaction between Cdc13 and Est1 (see **Figure 2**). Binding of MRX and reduced levels of Rif2 at short telomeres allows the recruitment of Tel1 that binds to the Xrs2 subunit of the MRX complex. Telomere recruitment of Tel1 strengthens the robust association of telomerase with telomeres through the phosphorylation of yet unidentified targets. In this model, we propose that following resection, a set of interactions between yKu, RPA, Est1, and Cdc13 prevent formation of telomeric ssDNA secondary structures and stabilize the telomerase holoenzyme at newly generated G-tail overhangs. *In vivo*, telomerase processivity may be positively regulated by Est1 and Est3, and also Tel1 (see text). G2-phase: after telomere elongation, telomerase is removed from telomeres as a result of the action of Pif1and probably through the action of phosphatases that restore the interaction of Cdc13 with Ten1 and Stn1. A cooperative interaction of Cdc13 with Pol1 and Stn1 with Pol12 recruits the pol alpha-primase complex to the telomeric ssDNA to direct the synthesis of the complementary C-strand. In this model, Rif1 assists in the synthesis of the C-strand and therefore counteracts telomere elongation by telomerase.

Another factor that can affect telomerase action is the repetitive nature and peculiar nucleotide composition of the DNA substrate itself. The G-rich single-strand 3′ overhang that constitutes the substrate for telomerase can adopt unusual secondary structures. In particular, it may fold into very stable G-quadruplexes or G4-DNA (Hayashi and Murakami, 2002; Zhang et al., 2010; Smith et al., 2011). These structures may constitute an obstacle for telomere replication, telomerase recruitment and telomere elongation by telomerase (Paeschke et al., 2010). Therefore, specific factors must exist to unwind G4-DNA either during semi-conservative replication, or after replication in order to maintain newly generated 3′ overhangs in a telomerase-extendible state (Gilson and Géli, 2007). Helicases such as Sgs1 and Pif1 constitute good candidates to unwind G-quadruplex structures at telomeres during telomere replication (Huber et al., 2002; Lopes et al., 2011; Paeschke et al., 2011). After semi-conservative telomere replication, Cdk1-regulated resection of blunt ends of leading strand telomeres generates G-tails whose lengths increase to about 50 nt during late S/G2-phase (Dionne and Wellinger, 1998; Frank et al., 2006; Vodenicharov and Wellinger, 2006). The timing of G-overhang lengthening is correlated with an enrichment of Cdc13 at telomeres although the increase of the number of Cdc13 molecules bound to the newly generated 3′ overhangs is difficult to estimate since Cdc13 binds also to the single-strand generated by semi-conservative replication (Faure et al., 2010). The Cdc13 mode of recognition of telomeric ssDNA indicates that at least 11 nt are required for full binding of Cdc13 (Hughes et al., 2000; Mitton-Fry et al., 2002). Thus, theoretically the transient 30–100 nt 3′ overhang can accommodate several Cdc13 molecules (Wellinger et al., 1993). However, ChIP experiments indicate that two other DNA-binding proteins, yKu and the ssDNA-binding protein replication protein A (RPA), also show an increased binding to telomeres by the time when telomerase is associated to telomeres (Schramke et al., 2004; Faure et al., 2010). The increase binding of yKu to telomere ends may reflect the release of yKu from TLC1 to the telomeric DNA when telomerase-bound yKu encounters telomeric DNA (see above). In its turn, RPA directly interacts with yKu and indirectly contacts telomerase (Luciano et al., 2012). In addition, the association of RPA with telomerase was shown to be dependent on both yKu and Est1 (Luciano et al., 2012). To reconcile these results, we propose that a cooperative action between yKu, RPA, Cdc13, and Est1 favors telomerase stabilization at telomeres through a set of protein interactions and through the prevention of secondary structure formation at telomeric G-overhangs (**Figure 1**).

## REGULATION OF ELONGATION AND PROCESSIVITY

In addition to its role in recruiting telomerase, Est1 appears to modulate telomerase DNA extension activity through a direct contact with Est2 (DeZwaan and Freeman, 2009). This hypothesis is consistent with the identification of *est1* alleles that reduce the activity of telomerase independently of its recruitment (Evans and Lundblad, 2002; Zhang et al., 2010). Similarly, Est3 that shares structural and functional similarities with TPP1 (Lee et al., 2008; Yu et al., 2008) interacts with the N-terminal (TEN) domain of Est2 and stimulates telomerase activity above basal levels *in vitro* (Talley et al., 2011). Hence, both telomerase-associated proteins Est1 and Est3 may influence telomere elongation by telomerase through interactions with the catalytic subunit Est2 in a way that remains to be elucidated (**Figure 1**).

**FIGURE 2:**
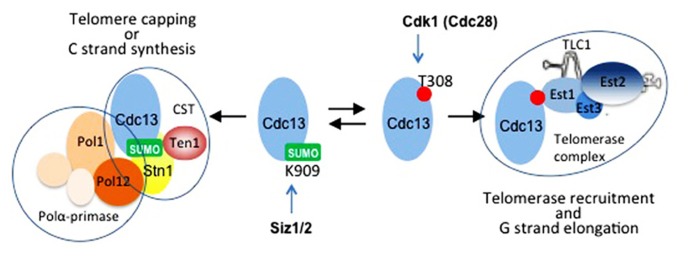
**Cdc13 at the crossroads.** Cdc13 forms separate complexes with different functions at telomeres. Cell cycle regulated post-translational modifications control the balance between these complexes. Siz1- and/or Siz2-dependent SUMOylation on Lys909 located in the C-terminus of Cdc13 promotes its interaction with Stn1 and formation of the CST complex. This modification peaks in early to mid S-phase (Hang et al., 2011). Cyclin-dependent kinase Cdk1 (Cdc28) phosphorylates Cdk1 on Thr308 located in the telomerase recruitment domain of Cdc13 Li et al., 2009). This modification occurs in late S to G2-phases, and it favors the interaction of Cdc13 with Est1 at the expense of its interaction with Stn1, thus promoting telomere uncapping and telomerase recruitment (Li et al., 2009). When telomerase action is accomplished, Cdc13 switches back to interaction with Stn1, and CST complex recruits polα-primase for the C-strand synthesis. Other modifications are likely involved in fine-tuning the balance between the complexes formed by Cdc13 (Wu et al., 2013).

These findings raise the question of the processivity regulation of the yeast telomerase that has been shown initially to be non-processive *in vitro* and *in vivo* (Prescott and Blackburn, 1997; Bosoy and Lue, 2004). An elegant study by Chang et al. (2007) in which they co-express two different telomerase RNA subunits shows that telomerase can dissociate and reassociate from a given telomere during one cell cycle. *In vivo* analysis also suggests that the yeast telomerase can be processive at extremely short telomeres (<125 bp; Chang et al., 2007). This result suggests a switch from a non-processive to a processive mode of telomerase action that has been proposed to depend on Tel1 although its precise mechanism remains elusive (Chang et al., 2007). One possibility would be that Tel1 facilitates the interaction between telomerase and its substrate to increase its processivity. As mentioned above, Tel1 is known to be recruited at short telomeres by virtue of its interaction with the Xrs2 subunit of the MRX complex. The Tel1 targets involved in both telomerase recruitment and telomerase processivity remain to be identified, although Cdc13, RPA, and the telomerase negative regulator Rif1 are potential candidates (Smolka et al., 2007; Luciano et al., 2012; Wu et al., 2013). Whether both Tel1-regulated pathways are the manifestations of the same process is an open question. Another related open question is whether Cdc13 moves with telomerase. In the processive mode, it is conceivable that Cdc13 remains bound to the telomerase complex as telomerase elongates the telomere and would ratchet in defined nucleotide steps as it has been proposed for POT1–TPP1 (Chen et al., 2012). In the non-processive mode, Cdc13 and the whole telomerase complex would dissociate from the 3′ overhang after a single round of addition of telomeric DNA repeats.

## A SWITCH FROM G- TO C-STRAND SYNTHESIS

The last step of telomere elongation is the synthesis of the complementary C-strand. As mentioned earlier, Cdc13 plays a role in the synthesis of both strands of the telomere, and by promoting the synthesis of C-strand, it limits continuous telomerase action (Grandin et al., 2000; Qi and Zakian, 2000; Chandra et al., 2001). A switch must operate that converts the Cdc13-telomerase complex into a CST complex allowing the efficient recruitment of the DNA polymerase alpha-primase to telomeres (Mason and Skordalakes, 2010; **Figure 1**). Recently, 17 novel *in vivo* phosphorylation sites were identified in Cdc13 (Wu et al., 2012). Intriguingly, several of these novel sites showed increased phosphorylation in G2/M making them good candidates to control this switch. However, how this switch functions and how it is coupled with the Pif1 catalyzed dissociation of telomerase from telomeres is still unknown (Boulé et al., 2005). Within the CST complex, the C-terminus of Stn1 has been shown to interact with both Cdc13 and Pol12 the B subunit of DNA polymerase alpha-primase complex (Puglisi et al., 2008). As a consequence, cells lacking the Stn1 C-terminus display an unusual telomere elongation phenotype yielding extremely elongated telomeres even in the absence of Tel1 function. Stn1 therefore provides a constitutive inhibitory signal, independent of telomere tract length, whose effectiveness is modulated by changes in its partner protein Cdc13 (Puglisi et al., 2008). Here again, the molecular details that govern the structural changes of Cdc13 and its associated proteins to balance the telomere association of telomerase and the polymerase α–primase are unknown. Rif1 whose deletion increases telomere length andis lethal for *stn1*Δ*C* cells may be a good candidate to govern this balance (Anbalagan et al., 2011).

## CONCLUDING REMARKS

Now that the main players in the telomere maintenance have been identified, the emphasis is given to the regulatory mechanisms that govern the transitions between the telomere states during the cell cycle. The challenge is to understand these transitions mechanistically, which will require structural information for the protein complexes as well as the complete knowledge of protein modifications and enzymes involved. The crucial targets of the Tel1, the major kinase responsible for telomere length homeostasis need to be identified. For the future, the main challenge would be to understand the telomere regulation in the context of chromatin and three-dimensional organization of the nucleus. Although the molecular details of the telomere regulatory pathways would likely differ between yeast and humans, the basic principles uncovered for a simple and tractable yeast system should provide guidance for understanding the complexity and finding better treatment for telomerase-positive cancers.

## Conflict of Interest Statement

The authors declare that the research was conducted in the absence of any commercial or financial relationships that could be construed as a potential conflict of interest.
